# Comprehensive epigenetic landscape of rheumatoid arthritis fibroblast-like synoviocytes

**DOI:** 10.1038/s41467-018-04310-9

**Published:** 2018-05-15

**Authors:** Rizi Ai, Teresina Laragione, Deepa Hammaker, David L. Boyle, Andre Wildberg, Keisuke Maeshima, Emanuele Palescandolo, Vinod Krishna, David Pocalyko, John W. Whitaker, Yuchen Bai, Sunil Nagpal, Kurtis E. Bachman, Richard I. Ainsworth, Mengchi Wang, Bo Ding, Percio S. Gulko, Wei Wang, Gary S. Firestein

**Affiliations:** 1 Department of Chemistry and Biochemistry, 9500 Gilman Drive, UC San Diego, La Jolla, CA 92093 USA; 2 Division of Rheumatology, Icahn School of Medicine at Mount Sinai, One Gustave L. Levy Place, New York, NY 10029 USA; 3 Division of Rheumatology, Allergy and Immunology, 9500 Gilman Drive, UCSD School of Medicine, La Jolla, CA 92093 USA; 4 Janssen Pharmaceuticals, 1400 McKean Road, Spring House, PA 19477 USA; 5 Department of Cellular and Molecular Medicine, 9500 Gilman Drive, UCSD School of Medicine, La Jolla, CA 92093 USA

## Abstract

Epigenetics contributes to the pathogenesis of immune-mediated diseases like rheumatoid arthritis (RA). Here we show the first comprehensive epigenomic characterization of RA fibroblast-like synoviocytes (FLS), including histone modifications (H3K27ac, H3K4me1, H3K4me3, H3K36me3, H3K27me3, and H3K9me3), open chromatin, RNA expression and whole-genome DNA methylation. To address complex multidimensional relationship and reveal epigenetic regulation of RA, we perform integrative analyses using a novel unbiased method to identify genomic regions with similar profiles. Epigenomically similar regions exist in RA cells and are associated with active enhancers and promoters and specific transcription factor binding motifs. Differentially marked genes are enriched for immunological and unexpected pathways, with “Huntington’s Disease Signaling” identified as particularly prominent. We validate the relevance of this pathway to RA by showing that Huntingtin-interacting protein-1 regulates FLS invasion into matrix. This work establishes a high-resolution epigenomic landscape of RA and demonstrates the potential for integrative analyses to identify unanticipated therapeutic targets.

## Abstract (web summary)

Fibroblast-like synoviocytes (FLS) in the intimal layer of the synovium can become invasive and destroy cartilage in patients with rheumatoid arthritis (RA). Here the authors integrate a variety of epigenomic data to map the epigenome of FLS in RA and identify potential therapeutic targets.

## Introduction

Rheumatoid arthritis (RA) is an aggressive immune-mediated joint disease with synovial inflammation and joint destruction^1^. Despite improvements in therapy, many patients have persistent inflammation and progressive disability. Epigenetic alterations such as DNA methylation and histone modification might contribute to RA pathogenesis and provide clues to identify novel therapeutic targets^2–7^. Much of the effort to define the RA epigenome has focused on fibroblast-like synoviocytes (FLS) of the synovial intimal lining, which invade the cartilage and assume a unique aggressive phenotype in patients with RA^8^.

Individual epigenetic marks, such as DNA methylation measured by arrays, have been investigated in RA, sometimes together with gene expression or DNA polymorphisms^9, 10^. The previous integrative analyses of these data were relatively simple, using overlapping marks to identify multi-evidence genes, for example. Despite insights gained from these analyses, a comprehensive and global characterization of the epigenomic landscape is needed to define its contribution to pathogenesis of RA. Furthermore, although, multiple omics technologies could provide a unique opportunity to define the global epigenomic landscape of RA, they also pose a great challenge to analyze in one integrative model. Segmentation methods, such as ChromHMM^11^ and Segway^12^ can identify functional elements but focus on histone modifications and have not incorporated other data such as DNA methylation. Furthermore, the epigenomic signals in RA and OA FLS mostly differ in magnitude rather than lead to distinct epigenetic states, which also limits the applicability of segmentation methods in which signals are discretized and the subtle amplitude difference diminishes. Therefore, a systematic approach is needed to integrate large-scale autoimmune disease epigenomes to define the epigenomic landscape.

In this study, we apply multiple omics technologies on primary cells from the site of disease in RA and control (osteoarthritis [OA]) FLS, including (1) chromatin immunoprecipitation followed by high-throughput sequencing (ChIP-seq) to map six histone modifications, (2) assay for transposase accessible chromatin with high-throughput sequencing (ATAC-seq) for mapping chromatin accessibility genome-widely, (3) RNA sequencing (RNA-seq) to measure whole genome RNA expression, and (4) whole-genome bisulfite sequencing (WGBS) for DNA methylation level quantification. To map the epigenomic landscape in RA, we integrate diverse epigenomic data into a single analysis using our unbiased method called EpiSig (http://github.com/Wang-lab-UCSD/EpiSig, see Supplementary Methods). This platform clusters regions with similar epigenomic profiles across all the RA and OA FLS samples. The epigenomic co-modifications identify clustered regions that share common functionality. As a result, this is the first time that histone modifications, WGBS, ATAC-seq and RNA-seq data have been combined into a single analysis to capture the deep complexity of the epigenomic landscape in any immune-mediated disease. Our approach incorporates signal intensities across all samples and can capture subtle epigenetic differences that distinguish RA. EpiSig clusters enriched with differentially modified epigenetic regions (DMER) were then extracted to identify RA-specific pathways and transcription factor motifs that could be mined for novel therapeutic targets. In addition to pathways known to be relevant to RA, unexpected ones also emerged. Of particular interest, the “Huntington’s Disease Signaling” pathway was discovered as highly significant and biologically validated. The new methodology and dataset provide a new way to identify RA-specific targets that can be used to develop novel therapeutic agents.

## Results

### Epigenomic landscape of RA and OA FLS

**Fig. 1:**
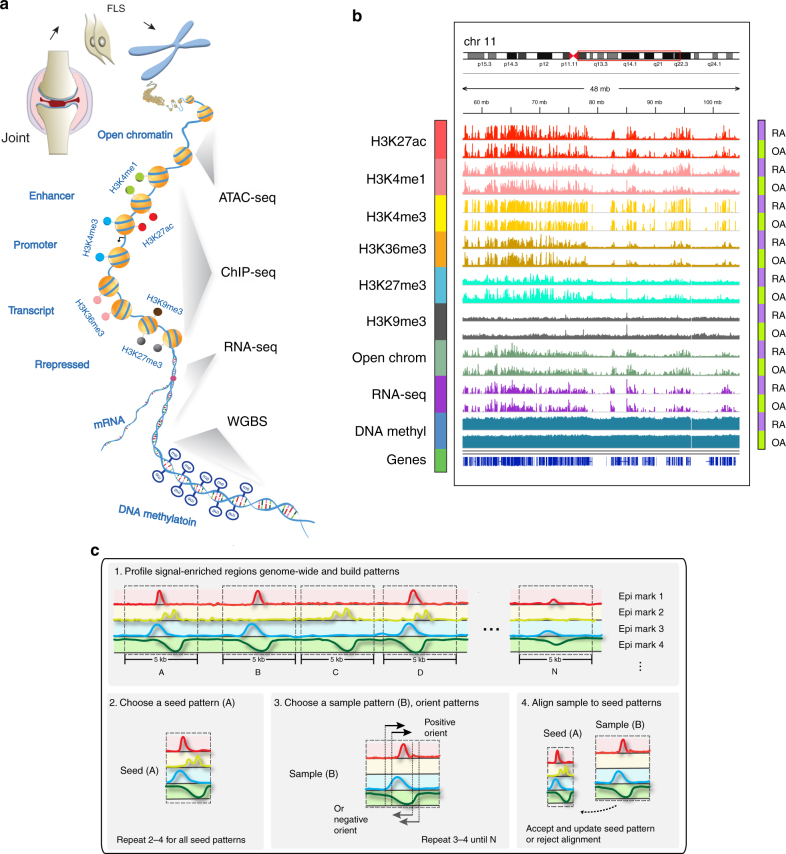
Overview of RA integrative analysis pipeline. **a** Schematic representation of major omics methods used in RA FLS. **b** Epigenomic landscape of RA FLS with six histone modifications, open chromatin, RNA-seq and DNA methylation. The figure shows an example of the relative signal intensity across a selected region of the genome for each mark in RA and OA FLS. In addition, the locations across chromosomes for the selected regions are indicated. **c** Schematic overview of how EpiSig integrates different epigenetic marks. The algorithm detects significant signals from sequencing data in 5 kb genome regions, clusters regions based on similar epigenomic patterns, and identify epigenomically similar regions

A total of 191 genome-wide datasets were generated across 11 RA and 11 OA samples, including 130 histone modification datasets, 22 open chromatin datasets, 20 RNA-seq datasets and 19 DNA methylation datasets. Six histone modifications marks were analyzed, including histone H3 lysine 4 trimethylation (H3K4me3), associated with promoter regions; H3 lysine 4 monomethylation (H3K4me1), associated with enhancer regions; H3 lysine 27 acetylation (H3K27ac), associated with increased activation of promoter and enhancer regions; H3 lysine 36 trimethylation (H3K36me3), associated with transcribed regions; H3 lysine 27 trimethylation (H3K27me3), associated with Polycomb repression; and H3 lysine 9 trimethylation (H3K9me3), associated with heterochromatin regions. Additional epigenomic marks include: open chromatin regions, profiled by ATAC-seq, denoting regions of accessible chromatin, and typically associated with regulator binding; DNA methylation, commonly associated with repressed regulatory regions, profiled with WGBS; and RNA-seq used for measuring gene expression levels (Fig. 1a). We integrated nine datasets and provided global views of the first high-resolution functional epigenomic landscape of RA and OA FLS (Fig. 1b).

### Identification of epigenomically co-modified regions

For integrative analysis of these diverse epigenomic data, EpiSig was used to find regions sharing similar epigenomic profiles across all the samples (Fig. 1c). The method first divided the whole genome into 5 kb regions and identified regions with any enriched epigenomic signals. We then iteratively identified clusters: in each iteration, regions that shared the most similar signal patterns were determined as a seed and the remaining regions in this cluster were assigned or rejected using a Gibbs sampling alike method. In each step, the 5 kb regions were shifted and oriented to align the patterns correctly. This is the first time WGBS, ATAC-seq and RNA-seq data have been integrated with histone marks to capture the greater complexity. The software is available at http://wanglab.ucsd.edu/star/EpiSig and http://github.com/Wang-lab-UCSD/EpiSig, and is further described in Supplementary Methods).

**Fig. 2:**
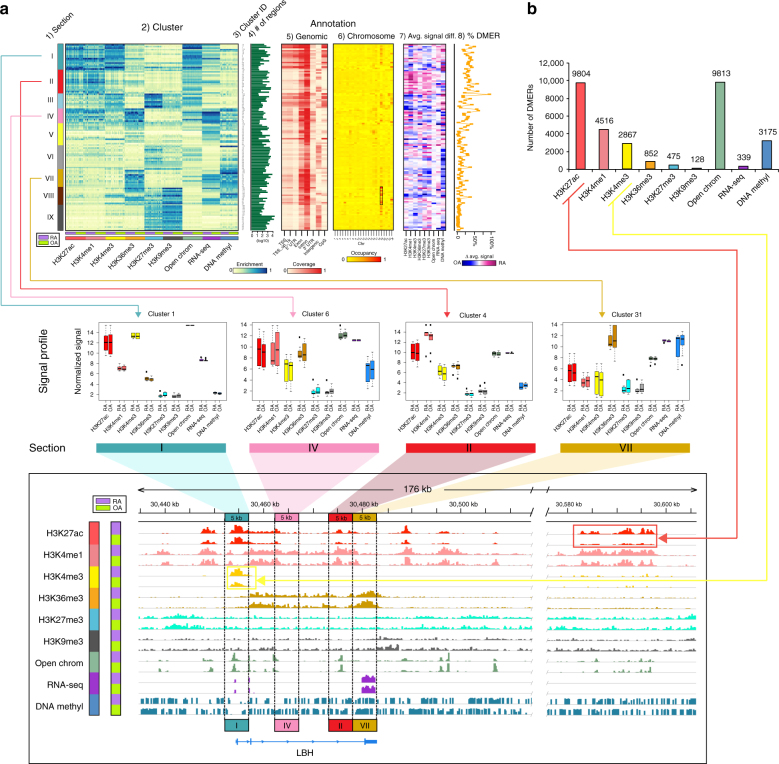
Genome-wide multidimensional clusters in RA and non-RA genomes. **a** A total of 125 genome-wide 5 kb multidimensional clusters were grouped into nine sections based epigenetic marks. From left to right of the upper panel shows the aligned 125 clusters, each of which includes 11 RA and 11 OA FLS lines: (1) the organization of clusters into epigenetically defined sections (see text); (2) genome annotation heatmap with coverage of each cluster, with 22 separate FLS lines shown within each cluster; (3) 125 cluster IDs; (4) the number of 5 kb regions in each epigenetic cluster (log10 scale); (5) the characteristics of each region within the clusters, such as the relative abundance of transcription start sites (TSS), gene bodies, or introns; (6) chromosome location of each region within the clusters (box shows high occupancy at chromosome 19); (7) differences in signals of epigenetic marks between RA and OA; and (8) percentage of DMERs each cluster. In the middle panel, boxplot examples of clusters in different sections corresponding to *LBH* regions in the genome browser (bottom panel) demonstrating differences between RA and OA and how the FLS marks can be categorized as sections I, II, IV, and VII^14^. **b** DMERs with enhancer and promoter histone marks account for the most common differences between RA and OA. The red and yellow lines connect H3K27ac and H3K4me3 bars to the genome browser, which shows an example of how those marks are differentially modified between RA and OA

EpiSig identified 169,502 signal-enriched 5 kb regions that were grouped into 125 epigenomic clusters. Each cluster contains an average of 1356 regions that share similar epigenomic profiles across all FLS lines (Fig. 2a and Supplementary Fig. 1). We then grouped similar epigenomic patterns (Fig. 2a). All the samples were sequenced together, so there was no batch effect that could contribute to these observations. To facilitate interpretation of the results, individual clusters were further combined into nine groups, which we call “sections”, using a self-organizing map algorithm (SOM).

Overall, the 9 sections show distinct epigenomically co-modified functional characteristics. Examples of these signal profiles in the epigenomic landscape are shown in Fig. 2a. Of the epigenomically defined sections, section I is highly enriched in H3K27ac, H3K4me3 and open chromatin regions with relatively weaker H3K4me1, which has high coverage in the TSS, 1 kb upstream of the TSS and 5′-UTR regions. CpG islands are also highly covered in this section, but most are de-methylated, indicating active promoter regions. Section II is enriched in H3K27ac, H3K4me1 signals and open chromatin regions, and low in DNA methylation, which is likely associated with active enhancer regions. This section is highly enriched with intergenic regions. Section III is largely composed of bivalent promoters, characterized by intermediate H3K4me3 signals together with the repressive mark H3K27me3, and enriched with regions around annotated TSSs. Notably, the poised promoters are highly enriched with chromosome X loci (~21% of regions in section III are located on chromosome X).

Section IV includes signals of H3K27ac and H3K4me1, together with strong signals of H3K36me3 and RNA-seq. In addition, this section covers low-intergenic regions and is likely associated with intragenic enhancers. Both section V and VII represent actively transcribed regions as indicated by strong H3K36me3 and RNA-seq signals, albeit relatively weaker in section V compared to section VII and with locations highly biased toward the gene body. Section VI and IX represent repressive regions marked by strong signal of H3K27me3. Compared with section VI, section IX shows a strong signal for heterochromatin mark H3K9me3 but much lower signals of open chromatin or DNA methylation. The coverage of intergenic region is high in these two sections. Lastly, H3K9me3 is enriched in section VIII together with high H3K36me3 signal, suggesting that these regions are associated with heterochromatin or zinc finger protein genes^13^. Interestingly, the genomic location of this section is largely biased toward chromosome 19 (35%) and X (35%) (Supplementary Fig. 2).

### DMERs between RA and non-RA

After defining the overall epigenomic landscape for FLS, we compared the relative intensity of each epigenetic mark between RA and our non-RA control. A total of 31,969 DMERs were identified across the 125 clusters (Fig. 2b and Supplementary Data 1). Open chromatin regions were found in most regions, with 9813 DMERs between RA and OA. A total of 339 differentially expressed genes (DEGs, >2-fold change and Benjamini-Hochberg [B-H] adjusted *p*-value <0.05) were also identified between RA and OA (Supplementary Table 1). Of these, 124 and 215 were over-expressed or under-expressed in RA relative to OA, respectively. For ChIP-seq DMERs, H3K27ac predominated in 9804 DMERs, followed by H3K4me1 and H3K4me3 with 4516 and 2867 DMERs, respectively (Supplementary Fig. 3). Compared to narrow peaks (H3K4me3, H3K4me1, and H3K27ac), histone marks with broad peaks (H3K27me3, H3K9me3, and H3K36me3) tended to have less peak abundance. In addition, 3175 DNA methylation DMERs were identified. Examples of H3K27ac and H3K4me3 DMERs are shown in the genome browser screenshot in Fig. 2a. The pairwise correlations between DMERs (histone modifications, open chromatin and DNA methylation) and gene expression are shown in Supplementary Fig. 4. Low correlations were observed for pairwise correlations, while the three-dimensional plots showed higher correlation among H3K4me1/3, RNA-seq and H3K27ac. To find the co-localization of DMERs and RA-associated genetic variations, 378 unique SNPs in 524 RA associations were downloaded from GWAS Catalog, 10 unique SNPs were overlapped with DMERs identified in our study (Supplementary Table 2). Interestingly, the co-localization was found in *LBH*^14, 15^, which was identified by our previous DNA methylation studies and plays a critical role in cell proliferation. It also provides further evidence that our unbiased methods have biologic relevance. The SNP associated with gene *FADS1, FADS2,* and *FADS3* overlapped with three DMERs (H3K27ac, H3K27me3, and H3K4me3).

### Integrative analysis reveals novel biological pathways

**Fig. 3:**
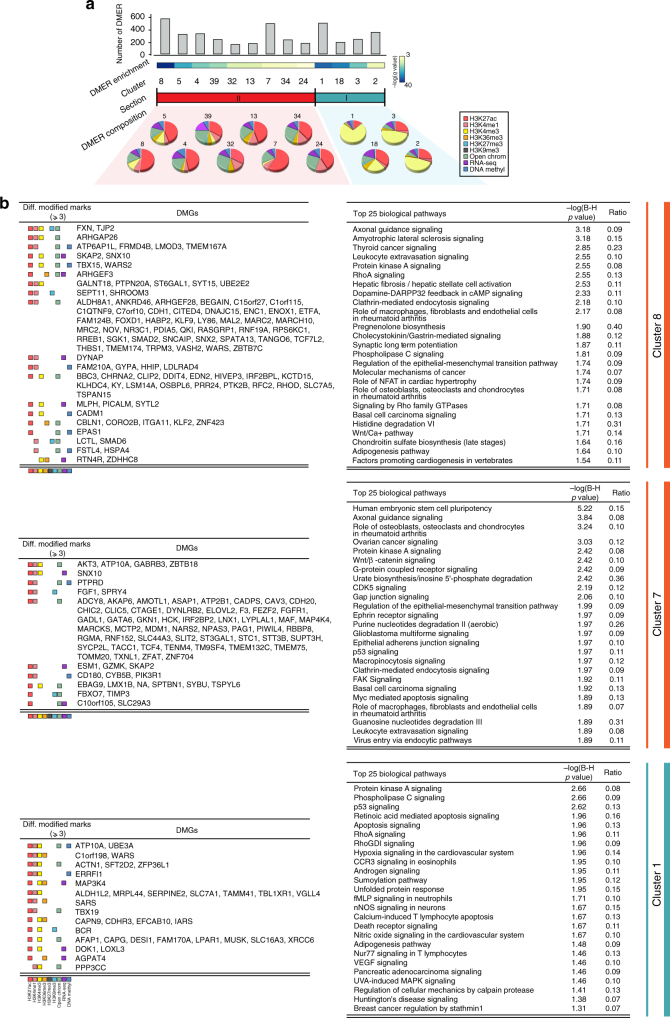
Integrative analysis identified 13 clusters with significantly enriched DMERs. **a** The 13 enriched clusters are shown with the number of DMERs in each cluster. Below, the relative abundance of various types of differential marks is shown and demonstrates the high abundance of active enhancer and active promoter marks (section I and II). Nine clusters are associated with active enhancers and four with active promoters. **b** Characterization of three clusters with the most DMERs (1, 7, and 8). Top DMGs (left) and top enriched biological pathways associated with these genes (right) are shown

To reveal the top enriched differentially modified biological pathways in RA, we used a stringent cutoff for DMERs (*q*-value <0.01) and selected 13 clusters with significant DMER enrichment for functional pathway analysis (Fig. 3a and Supplementary Table 3). Of these, nine clusters that associate with active enhancer regions had predominant differences in the number of H3K27ac DMERs (44.3% of the DMERs) that distinguish RA from OA (see Fig. 3a for the specific clusters). Moderate differences between RA and OA were also observed in open chromatin (21.1% of the DMERs). 8.4% of the differences between RA and OA in these clusters were due to the H3K4me1. The other four clusters had characteristics of active promoter regions, with large differences between RA and OA observed in mark H3K4me3 (47.4%) and intermediate differences in H3K27ac (25.1%). H3K4me1 DMERs account for 2.7% of all DMERs between RA and OA in these clusters. Thus, the key differences that distinguish the RA epigenomic landscape related to transcriptional regulatory regions like enhancers and promoters. The size, percentage and significance of enriched DMERs are shown in Fig. 3a.

Statistically significant differentially modified pathways (B-H adjusted *p*-value from Fisher’s exact test <0.05) involving the genes in the clusters with DMERs were found in 8 of the 13 clusters (Supplementary Table 4). Multiple enriched pathways in each individual cluster involved inflammation, immune response, matrix regulation, and cell migration, which supports the different known disease mechanisms of RA. Equally striking, however, was the number of biological pathways that could not be predicted based on the literature or current understanding of RA (see below). Cluster 8, with 579 DMERs, showed the highest DMER enrichment (Fig. 3a). Cluster 8 is associated with active enhancer regions, and 52% of the DMER differences between RA and OA are due to the H3K27ac mark. DMERs in open chromatin regions and DEGs are 16% and 10% of the DMERs, respectively. The cluster with the next most significantly enriched DMERs is cluster 1, which is associated with active promoter regions. About 74% of difference between RA and OA in cluster 1 is due to promoter mark H3K4me3. The activation mark H3K27ac accounted for ~11% of DMERs. Cluster 7 is the third most DMER enriched cluster and with a 59% difference between RA and OA for H3K27ac.

Interestingly, RA-specific pathways “Role of Osteoblasts, Osteoclasts and Chondrocytes in Rheumatoid Arthritis” and “Role of Macrophages, Fibroblasts and Endothelial Cells in Rheumatoid Arthritis” were significant and only enriched in clusters associated with active enhancer regions. Pathways enriched in clusters associated with active promoter regions are more generally associated with regulatory function. For instance, clusters 7 and 8, associated with active enhancers, are especially important as “RA-specific” clusters and have the two enriched RA-associated pathways (Fig. 3b and Supplementary Table 4). Therefore, these active enhancer associated regions are a characteristic chromatin state that distinguishes RA and OA.

Other notable pathways that have been observed in our previous integrative analysis^5, 16^ using less robust methodology and different technology (e.g., methylation chips) include “Integrin Signaling”, “Leukocyte Extravasation Signaling”, and “p53 Signaling”. The current dataset confirm those findings, validating the current methodology. As noted, many additional non-obvious pathways also emerge from the epigenomic landscape and could be used to identify potential pathogenic genes.

**Fig. 4:**
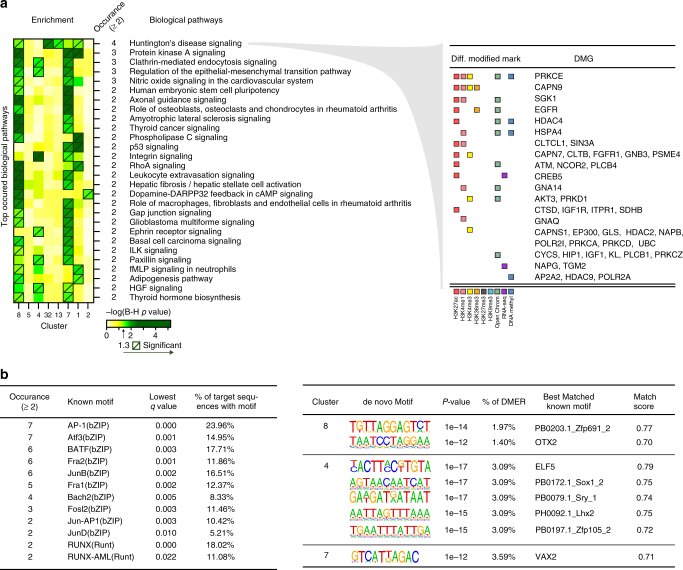
Prioritized enriched biological pathways and transcription factor (TF) motifs analysis. **a** Prioritized biological pathways ranked by the number of occurrences in the eight clusters with significantly enriched pathways (left) and DMGs with associated DMERs in “Huntington’s Disease Signaling” pathway (right). **b** Prioritized TF motifs ranked by the frequency in 13 clusters (left). The significantly enriched de novo TF binding motifs are shown on the right

To select biological pathways for subsequent target identification, pathways were also prioritized based on their frequency in the 8 clusters with significantly enriched pathways, and top 28 most frequently enriched ones that appeared in two or more clusters are shown in Fig. 4a. Among the most significant pathways, we found that “Phospholipase C Signaling”, “p53 Signaling”, “Integrin Signaling”, and “Protein Kinase A signaling” were particularly notable. Of interest, the  one that appeared the most often was totally unexpected, namely “Huntington’s Disease Signaling”. This pathway is enriched in four different clusters with 45 differentially modified genes (DMGs) associated (Fig. 4a) (see below for biologic validation).

### Cluster-specific transcription factor motif discovery

Motif analysis in DMERs regions was then performed on the 13 clusters with significantly enriched DMERs in order to understand the drivers of pathogenic pathways. Known motifs and de novo motifs that appeared in two or more clusters are listed in Fig. 4b. The top abundant known motifs are from bZIP and Runt families, binding to promoter regions to control gene expression. For example, TF AP-1 in bZIP family, containing c-Jun and c-Fos, regulates gene expressions of inflammatory cytokines, growth factors, and matrix-degrading matrix metalloproteinases (MMPs), and is crucial to joint destruction in RA^17^. The Runt family potentially plays a role in development of autoimmunity in RA. Interestingly, multiple transcription factors from Stat family are enriched in cluster 7 (Supplementary Table 5). One of these, STAT3, is a key pathway for IL-6 signal transduction, and IL-6 blockade is a highly effective therapy for RA and acts by blocking synovial STAT3 phosphorylation^18^.

### Biological validation of differentially modified pathways

To determine the functional relevance of non-obvious pathways, we focused on the “Huntington’s Disease Signaling” pathway because it was the most commonly enriched pathway and was not known to be associated with immune-mediated disease (Fig. 4a and Supplementary Fig. 5). There are a multitude of genes in this pathway that could serve as potential targets in this pathway, and we noted that on Huntingtin-interacting protein-1 (HIP1) appears in many key locations. The protein participates in clathrin-mediated endocytosis and cell signaling. It also regulates cell invasion in oncology^19, 20^ but its potential role has not been explored in inflammatory diseases.

**Fig. 5:**
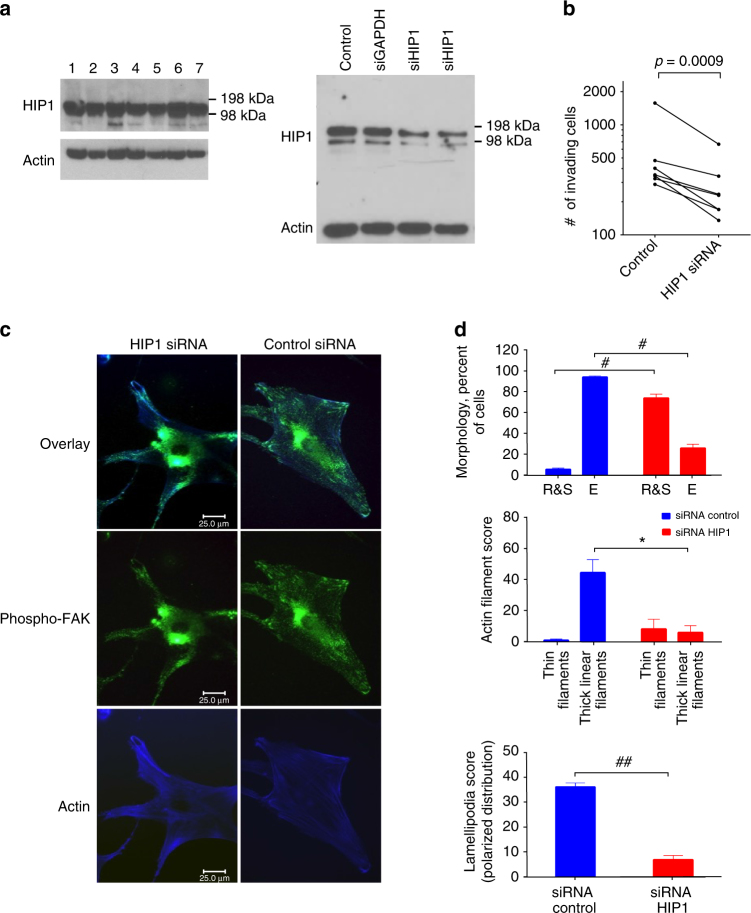
Biologic validation of HIP1 as a key pathway in RA FLS. **a** Left: HIP1 protein expression in FLS; right: decreased HIP1 expression induced by siRNA knockdown. **b** HIP1 deficiency significantly reduced RA FLS invasion through Matrigel. **c** HIP1 and cytoskeletal rearrangement. Left three panels: morphologic differences in HIP1 deficient cells demonstrate a stellar morphology with reduced number and size of lamellipodia and reduced co-localization of phospho-FAK. Right three panels: RA FLS transfected with siRNA control (green = phospho-FAK; blue = phalloidin/actin filaments; ×600 magnification). **d** Altered phenotype of the HIP1-deficient FLS. Top panel: siRNA HIP1 had reduced cell elongation (E elongated; R&S round and stellar morphology; #*p*-value = 0.0006). Middle panel: siRNA HIP1 decreased thin central filaments and thick actin filaments. *p-value = 0.003). Bottom panel: siRNA HIP1 knockdown also reduced the number of lamellipodia and their polarized distribution. (##*p*-value = 0.0008). All *p*-values are calculated by paired *t*-test, and error bars indicate standard deviations

Initial studies showed that HIP1 protein is constitutively expressed in FLS and can be depleted in cells with siRNA (Fig. 5a and Supplementary Fig. 6). We then examined the effect of control and HIP1-deficient FLS in an in vitro invasion model, a model that correlates with in vivo cartilage and joint damage^21, 22^. HIP1 deficiency unexpectedly decreased RA FLS invasion by nearly 50% (*n* = 7 separate FLS lines, *p*-value <0.001 (paired *t*-test), Fig. 5b). Morphology of HIP1-deficient RA FLS was then evaluated by confocal microscopy (Fig. 5c, d). HIP1 was depleted using siRNA, which decreased expression of the 116 kDa band on western blot but not other control proteins. HIP1-deficient cells lost their fusiform morphology and assumed a ‘stellate-like’ shape. Actin fibers became less organized and lamellipodia-like structures did not form or were present in a non-polarized manner without phospho-FAK co-localization. This phenotype is commonly observed in cells that cannot migrate or invade and suggests that HIP1 plays a role in FLS-induced invasion into cartilage in RA^23^.

## Discussion

Although treatment of RA has improved dramatically in recent years, current targeted therapies usually fail to induce remission and many patients do not respond to any available agent. Most efforts to develop new therapies rely on candidate gene approaches that have emerged from traditional biological research that dissect pathogenesis. This approach has limited potential to discover entirely new and unanticipated pathogenic processes that contribute to disease. To address this need, we developed genome-wide unbiased methodology and datasets that can identify surprising pathways and genes involved in immune dysfunction. By integrating multiple epigenetic technologies we hoped to define pathogenesis of disease and discover non-obvious targets.

The present studies focused on RA FLS which, unlike OA or normal synoviocytes, display an imprinted aggressive phenotype and invade cartilage explants when implanted in immunodeficient mice^24^. RA FLS can grow under anchorage-independent conditions, are less susceptible to contact inhibition, and are resistant to apoptosis^25, 26^. They are more invasive in vitro and produce cytokines and MMPs that serve as amplifying disease mechanisms^27^. Imprinted RA FLS can also migrate from joint to joint, potentially serving as a vector to disseminate the disease^28^. While the adaptive immune system clearly contributes to RA^17^, innate immune functions regulated by FLS also participate^29^ but have not been successfully targeted in humans.

We generated the largest collection of epigenomes for RA FLS by profiling six histone modification patterns, open chromatin regions, RNA expression and whole-genome DNA methylation and established the first high-resolution global epigenomic landscape for RA. To address the complex multidimensional relationships of those epigenomes and identify co-modified regions, integrative analysis was performed using a novel unbiased method, EpiSig. The analysis grouped the RA genome into 125 clusters based on epigenetic marks in multiple regulatory/functional elements. This is the first time whole-genome DNA methylation, chromatin accessibility, and the transcriptome have been incorporated into the integration method with histone modifications which allows a detailed understanding of the epigenomic landscape.

Our systematic integrative analysis identified 13 of the 125 clusters with statistically significant DMER enrichment for pathway analysis, which allowed us to prioritize biological pathways featured in promoter and enhancer regulatory modules. A variety of pathways were found, some of which were expected because they are related to immune responses or matrix regulation or were previously identified by our previous limited analyses. However other pathways were totally unexpected, such as “Huntington’s Disease Signaling” and a variety of others that still need to be explored. In the “Huntington’s Disease Signaling” pathway, many genes that could serve as potential targets. Previous studies found HIP1 is associated with cell movement in cancer^19, 20^. However, HIP1 is particularly prominent in this pathway and its potential involvement in oncology led us to reason that this might be especially interesting and worth exploring further. The fact that we were able to validate the role of a key protein in the “Huntington’s Disease Signaling” pathway indicates that our unbiased method can identify novel potential therapeutic targets. Other genes that are differentially marked in this pathway include phosphatidylinositide 3-kinases, activator protein-2, histone deacetylases, and heat shock protein-70. We are particularly intrigued by the potential use of histone deacetylase inhibitors to remodel the epigenome and alter enhancer activity, thereby modifying aggressive RA FLS behavior. In addition, transcription factor motifs were identified that distinguish RA from OA and the bZip motifs were particularly noted as enriched in DMERs. These motifs play a critical role in the regulation of metalloproteinases and cytokines that are pathogenic in RA.

Because, we have previously evaluated joint location-specific changes in RA^16^, we also assessed the differences between hip and knee FLS lines (*n* = 5 and 3, respectively for WGBS data). However, the present study was underpowered due to limited sample size and identified <200 DMERs using the same criteria. The previous studies showed that the magnitude of the differences between joints within RA is much less than the difference between OA and RA. Thus it is not surprising that many more samples are required to evaluate more subtle joint location-specific marks, and it is unlikely that these modest differences affect the overall RA vs. OA analysis.

The use of FLS permitted evaluation of a homogeneous cell population of primary cells from the site of disease and avoided the confounding problem of deconvoluting lineage-specific marks in mixed cell populations. The comparators for this analysis were OA FLS, which are derived at the time of joint replacement and are a commonly used comparator for RA because matched normal synovial cells are not readily available. We have previously shown that analysis of OA FLS DNA methylation patterns do not identify any systematic differences compared to normal that would affect the present study^4^. The use of cultured cells rather than freshly isolated cells could potentially influence the results, but we have previously shown that the DNA methylation signature is remarkably stable in RA FLS for many months in culture and they retain the aggressive phenotype^4^. Despite these potential limitations, the present studies provide a full dataset required for detailed analysis of primary cells isolated directly from the site of this destructive disease.

Our methodology focused on RA, but it is disease agnostic and can be applied to any immune-mediated disease if the datasets are available. The use of FLS simplified our studies because they are a homogenous cell lineage and play a critical role in rheumatoid joint damage. Studies on peripheral blood cells or other cells in disease tissue would require separation into individual lineages because single cell assays are not feasible with current technology for most of the marks studied. Future studies are required to validate the large number of unanticipated targets and pathways as well as explore diversity between patients and individualize treatment.

## Methods

### Human FLS

This study was approved by the Institutional Review Board of University of California, San Diego, and informed consent was obtained from all participants. Synovial tissue was obtained from patients with RA and OA at the time of total joint replacement or synovectomy. The diagnosis of RA conformed to American College of Rheumatology 1987 or 2010 criteria^30, 31^, and the diagnosis of OA conformed to American College of Rheumatology 1986 or 1991 criteria^32, 33^. The mean ages of RA and OA patients were 55 ± 9 and 66 ± 12, respectively. Joint locations were available for 10 RA patients (6 knee, 3 hip, 1 hand) and 11 OA (8 knee, 3 hip). Of the 7 RA patients with clinical information available, treatment included NSAIDs (*n* = 5), low dose prednisone (<5 mg/d) (*n* = 4), methotrexate (*n* = 4), methotrexate plus leflunomide (*n* = 1), TNF blocker (*n* = 3), IL-6 blocker (*n* = 1). Biologics, methotrexate and leflunomide were generally discontinued 4 weeks before surgery. Serology was available for 3 RA patients. One patient was RF positive and 2 patients were CCP and RF negative. Therapy information was available for 5 OA patients, who were treated with NSAIDs (*n* = 2) or analgesics. Enzymatic disaggregation of synovium was performed as previously described^14^. Cells were allowed to adhere overnight and non-adherent cells were removed. Adherent FLS were split at 1:3 when 70–80% confluent and used from passages 4 through 7, when they are a homogeneous population of fibroblasts. Primary FLS were cultured (at 5% CO_2_, 37 °C) in Dulbecco’s modified Eagle’s medium (DMEM) supplemented with l-glutamine, gentamicin, penicillin/streptomycin, and 10% heat-inactivated fetal bovine serum (FBS) as previously described^34^. For synchronization experiments, cells were serum starved for 2 days and transfected with siRNA in DMEM containing 1% FBS and supplements, followed by addition of 10% FBS.

### RNA-seq data processing

Total RNA was extracted and the quality of all samples was evaluated using an Agilent Bioanalyzer. The samples had an average RNA Integrity Number (RIN) of 9.4 with a minimum of 7.5. Sequencing libraries were prepared using TruSeq Stranded Total RNA RiboZero protocol from Illumina. Libraries were pooled and sequenced with an Illumina HiSeq 2000. Raw read quality was evaluated using FastQC.

Adapter and low quality bases below a quality score of 15 were trimmed from raw RNA-seq reads. After trimming, reads with less than 30 bp were further discarded. The remaining reads were aligned to human reference genome hg19 using STAR (2.3.0) and assembled and quantified by HTSeq (0.5.4p5). DEGs were identified using DESeq2 package in R. To be considered a DEG, twofold change of gene expression levels between RA and OA should be achieved and the B-H adjusted *p*-value is <0.05. For the following analysis, transcription levels were then converted to log2 of the normalized counts.

### ChIP-seq data processing

The sample preparation for ChIP-seq was performed using the Zymo-spin ChIP kit (Zymo Research, Irvine, CA) according to the manufacturer’s instructions. Raw ChIP-seq reads of 11 RA and 11 OA samples, each with six histone modifications (H3K4me1, H3K4me3, H3K9me3, H3K27ac, H3K27me3, and H3K36me3) and input data, were mapped to hg19 genome using bwa (0.7.7-r441). Peaks were called using MACS2 (2.1.0.20150420.1) using a *p*-value cutoff of 0.1 for broad marks (9me3, 27me3, 36me3) and 0.01 for narrow marks (4me1, 4me3, 27ac). To assess the library complexity and the enrichment of the data, quality control was done using several metrics, including non-redundant fraction of reads, fraction of reads that fall into peak regions, PCR bottleneck coefficient, normalized strand cross-correlation coefficient and the relative strand cross-correlation coefficient. ChIP-seq DMERs were determined by DiffBind package in R with *q*-value <0.05 for six marks.

### ATAC-seq and WGBS data processing

ATAC-seq sample preparation was followed by previous protocol^35^ and was sequenced by Epinomics (San Francisco, CA). ATAC-seq data were processed using Bowtie and read depth was normalized. ATAC-seq DMERs were determined by DiffBind package in R with *q*-value <0.05. The bisulfite conversion and sequencing were performed by GATC Biotech AG (Germany). WGBS data were processed with DSS package in R. Differentially methylated loci (DMLs) were determined and Differentially methylated regions were defined by callDMR function with delta beta of DMLs >0.05 and *q*-value <0.0015.

### Genome-wide multidimensional clustering by EpiSig

EpiSig is derived from the previously published method ChromaSig^36^ with greatly improved performance and capability in capturing sequencing profile patterns among large number of datasets. Compared to previous ChromaSig developed in Perl script, EpiSig was rewritten with C++ in a single standalone program and provides two running modes, which significantly improved speed and performance. Regarding to searching accuracy, EpiSig is an unsupervised learning approach to simultaneously clusters, aligns sequencing patterns without prior knowledge. Before the pattern alignment, one important step is to find the initial seeds among the remained loci. EpiSig provides another information entropy based method to find the candidate seed loci. First, the maximum intra-entropy loci (enriched signal window) are selected, then the minimum inter-entropy loci are added to the seed set. Actually they measure the similarities among them. To handle the different sequencing types such as histone modification data, RNA-Seq data and other data types, EpiSig utilizes a bi-clustering thinking to concatenate different data types into one larger data window when aligning them. This provides a more meaningful and accurate pattern discovering approach.

To assess the co-modified regions in RA and non-RA, all sequencing datasets were processed, normalized and filtered ensuring a uniform description of the information based on read depth. For final data processing, inputs were subtracted from the histone mark data. The comprehensive dataset is then preprocessed by EpiSig by dividing the data into binned binary files. After this, EpiSig performs data normalization using a sigmoid function, followed by scanning the data for enriched regions. To facilitate interpretation of the result, these individual EpiSig clusters were further combined into sections using a SOM algorithm.

### Genome and chromosome annotation of targeted regions

The human genome hg19 was used as reference genome with functional annotation in GENCODE v19. CpG islands and TSSs were downloaded from UCSC table browser. The genome annotation was performed by overlapping targeted regions with gene exon, intron, intergenic region, 3′- and 5′-UTR, TSS, 1 kb upstream of TSS, and CpG island. The coverage is defined as the percentage of overlapped regions. The chromosome annotation was calculated as the percentage of 5 kb regions in each chromosome and followed by chromosome length and cluster size normalization.

### Pairwise correlations of DMERs and gene expressions

To plot correlations between DMERs and gene expressions, DMERs (from six histone modifications, open chromatin regions, and DNA methylation) were assigned to nearby genes using GREAT analysis with default settings^37^. If multiple DMERs were assigned to the same gene, the average of fold changes was calculated. Log2 fold change was used for the plot except for WGBS.

### Pathway prioritizing

To prioritize enriched pathways, stringent cutoff of *q*-value <0.01 was applied to DMERs from six histone modification marks and open chromatin region. Because different analysis methods were used for identifying DEG and DNA methylation DMERs, no additional filter was applied. Enriched DMERs (from six histone modifications, open chromatin regions and DNA methylation) were assigned to nearby genes using GREAT analysis with default settings. The DMER assigned genes together with DEGs were collected for pathway analysis in each cluster. However, we did not assign different weights to DEGs/DMERs. To test for significant enrichment of DMER in each cluster, hypergeometric test was performed using R package. For prioritizing pathways, clusters with hypergeometric test *q*-value <0.05 and number of associated genes >200 were selected. Ingenuity pathway analysis (IPA) (QIAGEN Redwood City) was used for pathway analysis. Finally, pathways were prioritized based on their frequency in the enriched clusters.

### Motif discovery

Motif analysis was performed in 13 clusters using HOMER^38^. Due to small input regions, *q*-value of 0.1 was used as the cutoff of known motif enrichment. de novo motif was also discovered and scores were calculated indicating matches to known motifs. de novo motifs with *p*-value <1e−12 and score >0.7 were listed.

### siRNA knockdown of HIP1

Dharmacon SMARTpool siRNA targeting HIP1, GAPDH, and a non-coding control were purchased from Thermo Scientific (Lafayette, CO) and transfected into RA FLS according to the manufacturer’s instructions. Briefly, FLS derived from RA FLS were transfected siRNA in the presence of DharmaFECT reagent 1 in DMEM media with NEAA. Two days later, cells were used for invasion experiments or immunofluorescence. Aliquots were used for cell lysis to confirm gene knockdown with western blot and qPCR.

### FLS invasion assay

Invasion was assayed in a transwell system using Matrigel-coated inserts (BD Biosciences, Franklin Lakes, NJ), as previously described^39, 40^. FLS in serum free medium were placed in the upper compartment of the Matrigel-coated inserts. The lower compartment was filled with 10% FBS and incubated for 24 h. The insert was stained with crystal violet and the total number of cells that invaded through Matrigel was counted with ImageJ cell counter software.

### Western blot analysis

A quantity of 5 μg of FLS protein/lane were loaded into a NuPAGE 10% Bis-Tris gel (Invitrogen) in the presence of MES buffer (Invitrogen) and electrophoresed under reducing conditions. After transfer, the membranes were incubated with anti-HIP1 rabbit monoclonal antibody (Abcam, Cambridge, MA). HRP-conjugated anti-rabbit IgG antibody (GE Healthcare, Pittsburgh, PA) was used as secondary antibody. Proteins were visualized with Clarity Western ECL substrate (Bio-Rad, Hercules, CA). Rat anti-actin antibody (Cell Signaling technology, Danvers, MA) was used as loading control.

### HIP1 gene expression

qPCR was performed as previously described^41^. Ct (threshold cycle) values were adjusted for Ubiquitin in each sample (ΔCt), and fold differences were calculated with the 2−ΔΔCt method.

### Confocal immunofluorescence microscopy

RA FLS were cultured on coverslips to 20–30% confluence, transfected for 6 h with HIP1 or control siRNA, serum starved overnight and then treated for 5, 15, 30 min with complete media with 10% FBS. Cells were then fixed with 4% formaldehyde for 15 min and permeabilized with PBS/Triton X-100, 0.1% for 5 min. Non-specific binding was blocked with 5% nonfat milk. A rabbit anti-phospho-FAK (Y397) (Abcam) was used as primary antibody, and a Alexa Fluor 488 (green) donkey anti-rabbit IgG (Invitrogen) used as secondary antibody. Alexa Fluor 350 (blue) Phalloidin (Invitrogen) was used to stain the actin filament. A Leica DMi8 fluorescent microscope system was used for visualization with the appropriate filters, with Leica Lax X software. Cell cytoskeleton was scored as previously described^39^ using a ×600 magnification.

### Data availability

The data that support the findings of this study have been deposited in Gene Expression Omnibus with the primary accession code GSE112658. Other data that support the findings of this study are available from the corresponding authors upon request.

## Electronic supplementary material

Supplementary Information

Description of Additional Supplementary Files

Supplementary Data 1
