# Supplementary material for: Comprehensive epigenetic landscape of rheumatoid arthritis fibroblast-like synoviocytes
Source: Nat Commun. 2018 May 15;9:1921. doi: 10.1038/s41467-018-04310-9 (PMC5953939; doi:10.1038/s41467-018-04310-9)
Supplement: Supplementary file 1 — Supplementary Information [file 41467_2018_4310_MOESM1_ESM.pdf]

Supplementary Information for

Comprehensive Epigenetic Landscape of Rheumatoid Arthritis Fibroblast-like Synoviocytes

Ai *et al.*

Supplementary methods

EpiSig

1) Data normalization

After performing QC, for the data preprocessing we first aligned the raw fastq files unique to the genome and filtered out all duplicate reads. We then read-depth-normalized the samples and normalized again using the input reads. Since a certain amount of regions can be problematic in terms of anti-body binding we excluded all encode blacklisted locations from the reads. These regions were often found at specific types of repeats (e.g. centromeres, telomeres etc).

Since each histone peak set can have its own min-max peak distribution and vastly varying signal-to-noise ratios we also performed a quantile normalization on the binned raw reads that belong to the same type of mark.

These normalized samples were uploaded into EpiSig, which processes the reads by first binning them into 100-bp bins, containing the number of read-counts in each bin. It then normalizes the number of reads for each mark by using a sigmoid function to account for the number of unique monoclonal reads as they may still be highly variable between different histone marks. Here, $x_{h,i}$ is the sigmoid function:

$$x'_{h,i} = \frac{1}{1 + e^{-(x_{h,i} - median(x_h))/std(x_h)}}$$

where median(x_h) and std(x_h) are the median and standard deviation of the number of tags in the 100-bp bins for histone mark h. In this scenario, bins will have a count of 0.5 for median number of reads.

2) Finding peaks

Locations within the samples that are significant are identified using χ^2 statistics for each locus of size w starting at the jth bin:

$$y_{h,j} = \sum_{k=1}^{w} z^2_{h,j+k} \sim \chi^2_w$$

for each of the marks separately. We use a *p*-value cutoff of 1e-5 for significance evaluation.

3) Self-organizing maps (SOM)

We observed that within our hierarchical clusters of the EpiSig clustering results, similar enrichment patterns did not necessarily group together as expected. For example, a similar pattern enriched with the same marks but slightly different enrichment in another mark would not be grouped next to each other. Even leaf sorting of the hierarchical clusters, while helping, did not give a satisfying order of the clusters of the heat map. We therefore used SOM to arrange the clusters. In our setup, the algorithm treats each enrichment row as a vector and then rearranges them into clusters by iteratively training on the data vectors. We used several settings for the number of nodes k and the iterations i. In our case, k=16 and a training iteration of i=500 gave us a converging plot of the variance of the training vectors, i.e the average quantization error of the training data vectors. Finally we plotted the new cluster-order and visually checked whether the clusters made sense by comparing the enrichments in each cluster group (CG).

Supplementary figures

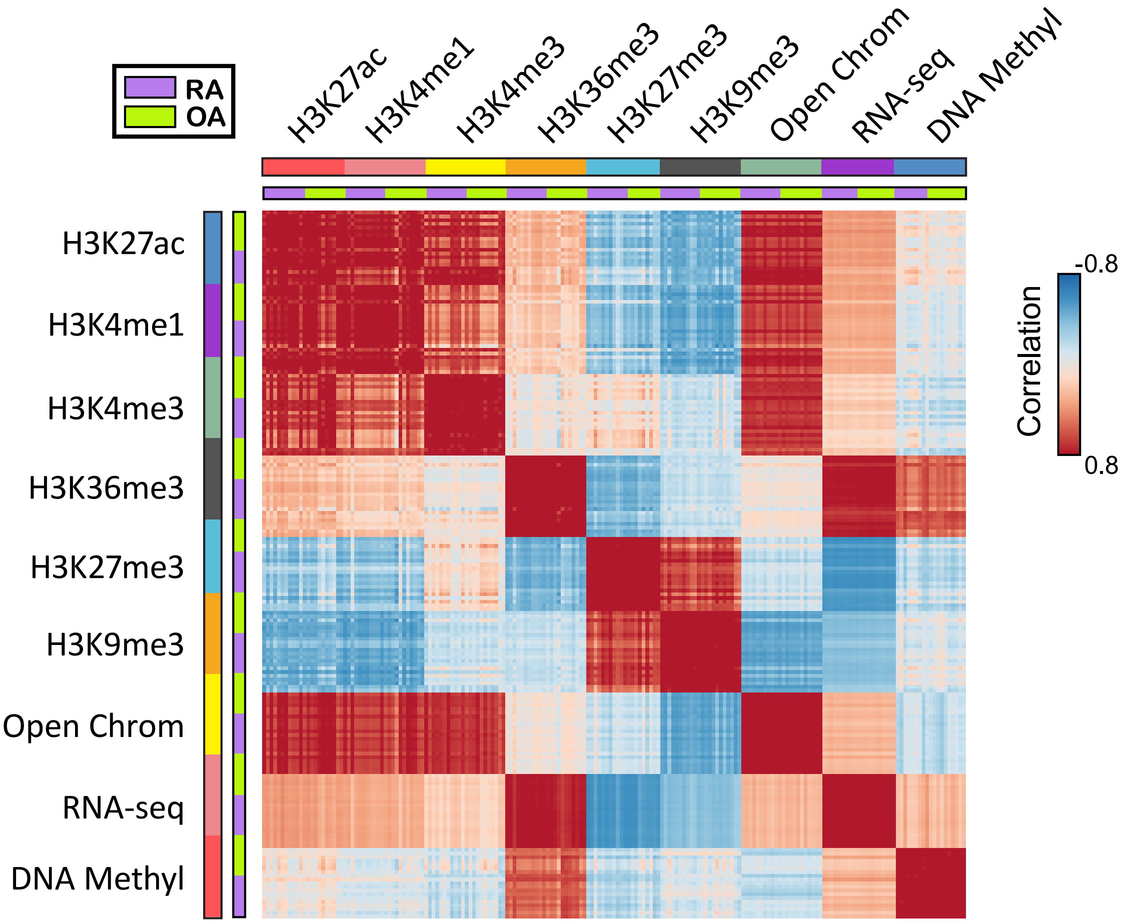

Supplementary Fig. 1. Pearson correlations of nine marks in 11 RA and 11 OA. H3K27ac, H3K4me1 and open chromatin marks are highly correlated, with weaker correlation with promoter mark H3K4me3. RNA-seq is highly correlated with gene body mark H3K36me3. DNA methylation is also positively correlated with H3K36me3. H3K9me3 is highly correlated with repressive mark H3K27me3, while these two marks are negatively correlated with almost all other marks.

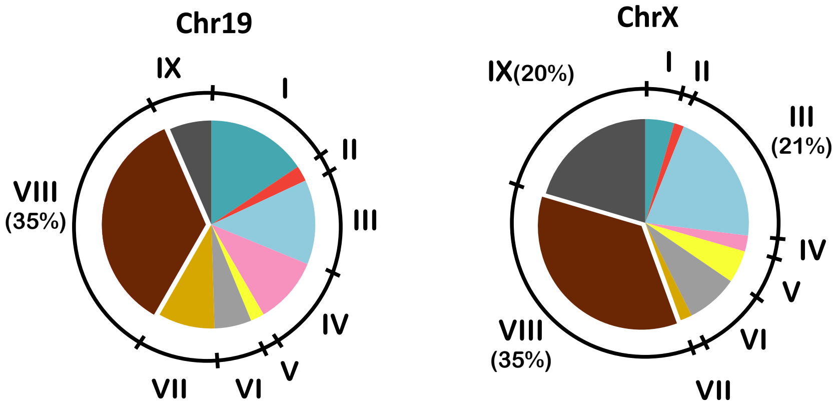

Supplementary Fig. 2. Pie charts show chromosome 19 and X occupancy in nine sections. *Section VIII*, associated with heterochromatin or zinc finger protein genes, has the largest genomic location occupancy biased on chromosome 19 (35%) and X (35%) compared to other sections. In addition, *section III* and *IX* have ~20% occupancy in chromosome X.

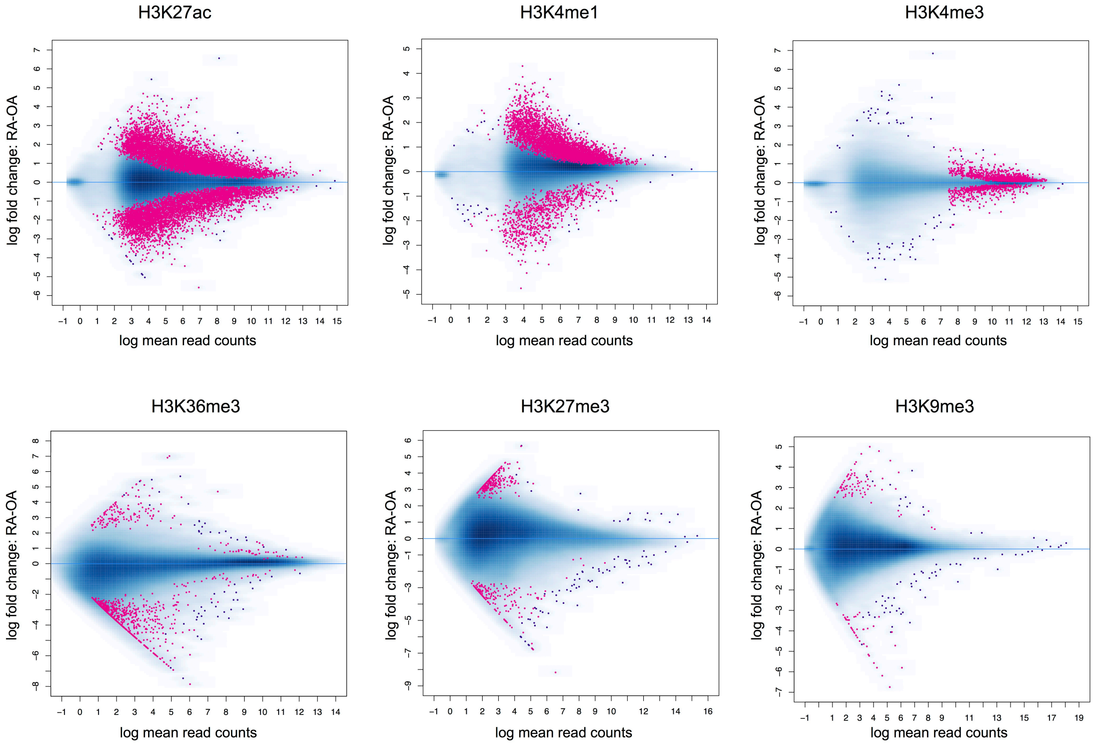

Supplementary Fig. 3. MA plots of histone modification level fold changes at individual DMER illuminated effect sizes of changes. Each point represents a region, with points in red representing DMERs (q-value < 0.05). Histone marks with narrow peaks in the upper panel (H3K27ac, H3K4me1 and H3K4me3) have more numbers of DMERs than those with broad peaks in the lower panel (H3K36me3, H3K27me3 and H3K9me3).

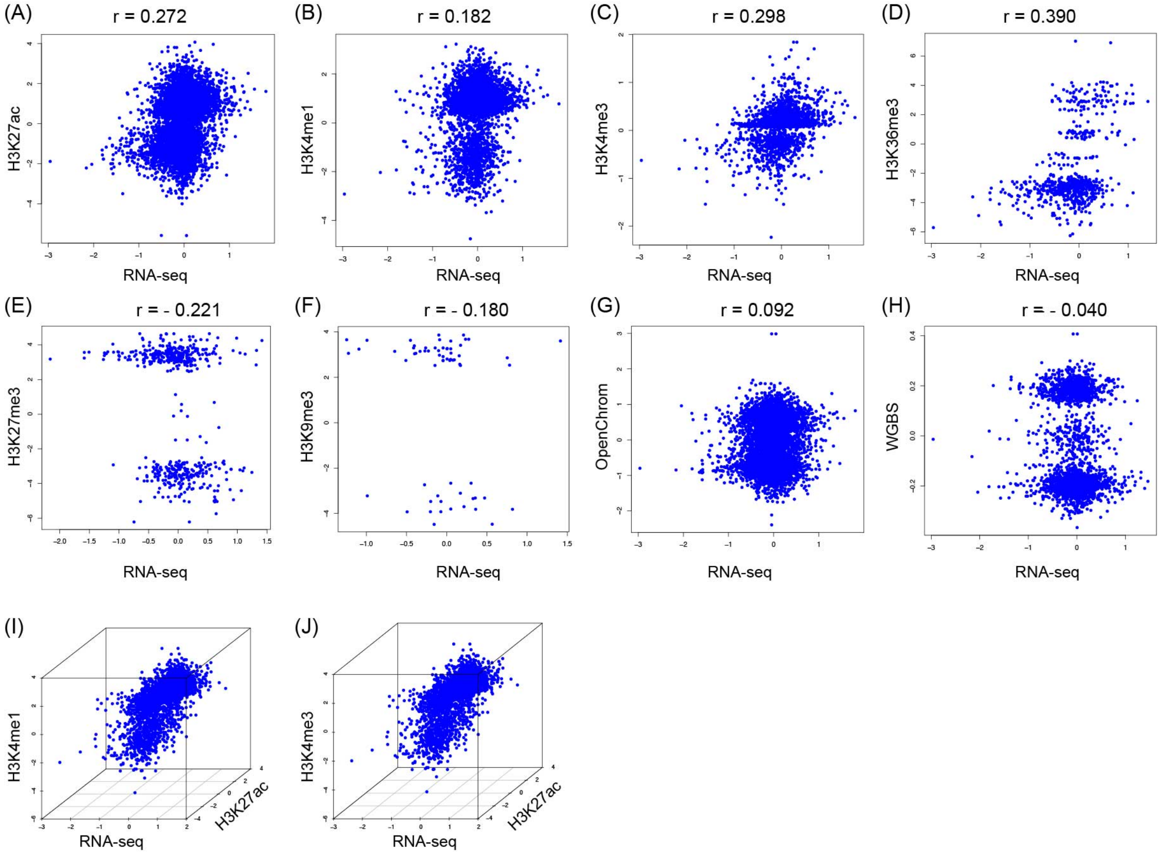

Supplementary Fig. 4. Correlations between DMERs and gene expressions. DMERs were assigned to genes with GREAT in default setting. Log2 fold change was used for the plot except for WGBS. (A-H) Pairwise correlation of epigenetic mark and gene expression with Pearson correlation coefficients displayed aboved. (I-J) Three-dimension plots show the correlation of H3K4me1/3, RNA-seq and H3K27ac.

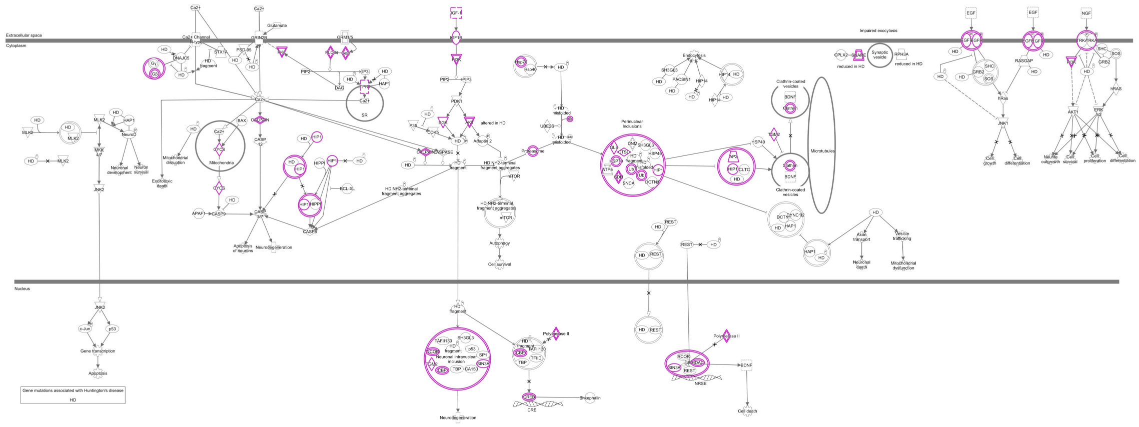

Supplementary Fig. 5. The ingenuity pathway of "Huntington's Disease Signaling". Differentially modified molecules are indicated in magenta color. Legends: ▽ Kinase; ◇ enzyme; ⬡ peptidase; ⏢ transporter; ⬯ transmembrane receptor; ⬭ transcription regulator; ▭ chemical; ▯ G-protein coupled receptor; ⬚ growth factor; ⬚ ion channel; ◯ other; double-sided shapes: complex/group. The pathway was generated through the use of QIAGEN's Ingenuity Pathway Analysis (IPA®, QIAGEN Redwood City, www.qiagen.com/ingenuity)

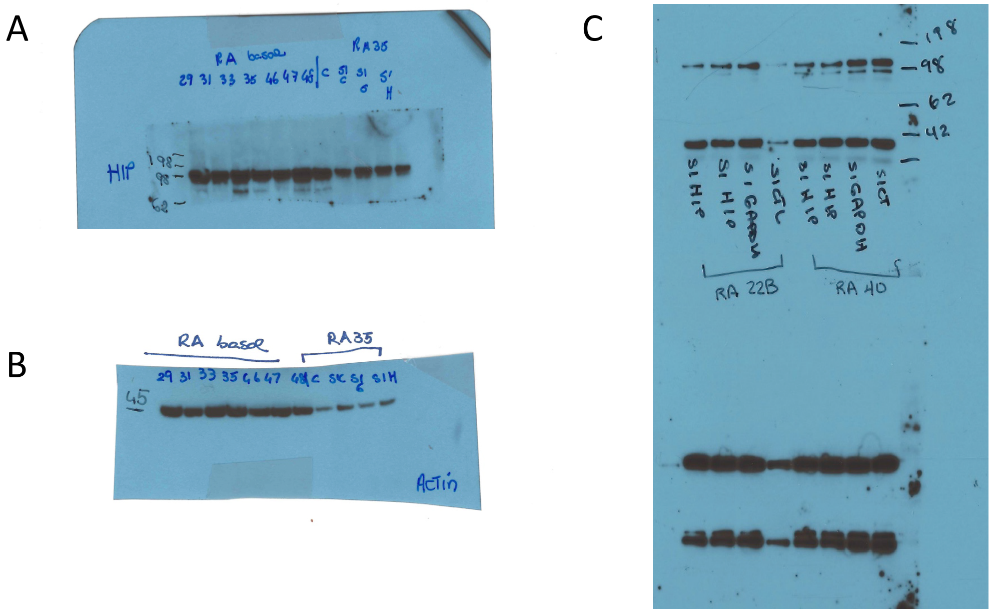

Supplementary Fig. 6. Original scans of Western blots with siRNA knockdown of HIP1 and actin. (A) HIP1 and **(B)** actin protein expression in FLS with molecular weight marker. **(C)** Decreased HIP1 expression induced by siRNA knockdown.

Supplementary tables

Supplementary Table 1. 339 DEGs between RA and OA by RNA-seq. (B-H adjusted *p*-values are calculated by DESeq2 package in R)

Gene	log2 fold change (RA/OA)	B-H adjusted *p*-value	Gene	log2 fold change (RA/OA)	B-H adjusted *p*-value	Gene	log2 fold change (RA/OA)	B-H adjusted *p*-value
CTD-2066L21.1	1.85	2.32E-15	*PROSC*	1.00	2.44E-02	*AKR1C2*	-1.03	5.38E-04
BDNF-AS	1.26	4.27E-09	*AC007255.8*	1.02	2.68E-02	*AC008964.1*	-1.42	5.42E-04
RP11-801F7.1	1.00	9.19E-09	*MOXD1*	1.04	2.78E-02	*PALMD*	-1.01	5.43E-04
SLC7A11-AS1	1.29	1.74E-08	*CTD-2313F11.1*	1.04	2.94E-02	*RP11-157F20.3*	-1.04	5.75E-04
SLC7A11	1.24	6.39E-08	*MIR3689E*	1.03	3.02E-02	*ATP8B4*	-1.35	5.89E-04
RP11-688I9.2	1.32	1.12E-07	*RPL17P49*	1.03	3.05E-02	*TYR*	-1.43	6.19E-04
RP11-133O22.6	1.60	3.96E-07	*RN7SKP275*	1.02	3.26E-02	*PGR*	-1.26	7.42E-04
CTD-2218G20.2	1.69	3.96E-07	*AL645728.1*	1.01	3.35E-02	*RP11-521C20.3*	-1.08	7.71E-04
CTD-3253I12.1	1.34	3.96E-07	*RP11-346D6.6*	1.01	3.48E-02	*KCNJ15*	-1.10	8.03E-04
AC073072.5	1.48	4.13E-07	*AC016909.1*	1.01	3.62E-02	*LPIN2*	-1.03	8.29E-04
RP11-627G18.1	1.27	9.80E-07	*F2RL2*	1.00	3.63E-02	*CLDN4*	-1.12	8.29E-04
SRGN	1.81	9.86E-07	*RP11-473L15.2*	-3.17	3.55E-36	*RP4-704D23.1*	-1.40	8.98E-04
NUDT6	1.04	1.42E-06	*FGF10*	-2.96	3.64E-25	*CTB-13F3.1*	-1.02	9.13E-04
BCL7A	1.24	1.63E-06	*RAB40A*	-1.58	5.31E-13	*AC013444.1*	-1.41	9.49E-04
INPP4B	1.40	2.85E-06	*RP4-718D20.3*	-2.26	5.31E-13	*STON2*	-1.40	9.78E-04
RP1-69D17.3	1.07	3.20E-06	*CTD-2515H24.4*	-2.05	5.84E-13	*BCL2L11*	-1.15	9.78E-04
RP11-287D1.3	1.00	3.33E-06	*RP11-737F9.1*	-2.46	3.31E-12	*AC073626.2*	-1.32	9.78E-04
AC012513.6	1.46	3.45E-06	*RP11-680F20.12*	-1.41	5.11E-12	*MORN4*	-1.13	9.81E-04
RP11-807H7.1	1.82	5.67E-06	*AC004540.4*	-1.84	6.79E-12	*EPB41L1*	-1.10	9.87E-04
KIRREL3-AS2	1.03	6.40E-06	*RP4-647C14.3*	-1.93	3.73E-11	*GPRASP1*	-1.17	1.01E-03
TNFRSF11B	1.55	8.17E-06	*SNX10*	-2.16	1.52E-10	*CACNA2D3-AS1*	-1.15	1.03E-03
FLG-AS1	1.78	8.18E-06	*DLX4*	-1.81	3.95E-10	*RPL5P11*	-1.06	1.07E-03
CTA-299D3.8	1.39	1.92E-05	*AC004543.1*	-2.23	2.89E-09	*SLC4A4*	-1.21	1.09E-03
RP11-314C9.2	1.27	2.07E-05	*CCDC41*	-2.04	3.04E-09	*RP3-512E2.2*	-1.16	1.18E-03
RP4-536B24.2	1.29	3.71E-05	*RP11-473L15.3*	-2.22	5.05E-09	*IL12A*	-1.21	1.23E-03
RP11-567G24.3	1.46	4.76E-05	*ADAMTSL1*	-1.64	9.86E-09	*ACOXL*	-1.37	1.35E-03
SLC38A1	1.07	5.55E-05	*PDGFRL*	-1.81	1.29E-08	*MINK1*	-1.26	1.55E-03
GCSAM	1.01	8.11E-05	*VIT*	-1.44	2.10E-08	*SUMF2*	-1.09	1.76E-03
RP11-373J21.1	1.35	8.32E-05	*CTD-2562J17.9*	-1.46	3.68E-08	*GRIA1*	-1.24	2.15E-03
AC002454.1	1.13	8.61E-05	*SLC7A8*	-1.57	1.10E-07	*ABCA8*	-1.17	2.41E-03
NR2F1	1.32	1.16E-04	*RP11-234K24.3*	-1.48	1.32E-07	*EPB41L4A-AS2*	-1.31	2.71E-03
NLGN1	1.32	1.17E-04	*CTD-2562J17.7*	-1.46	1.71E-07	*PSCA*	-1.11	2.73E-03
NLGN1-AS1	1.27	1.17E-04	*RP11-290H9.4*	-1.54	2.66E-07	*PRKAG3*	-1.13	2.79E-03
EPHB1	1.29	1.27E-04	*RP11-624G17.3*	-1.80	3.08E-07	*PPP1R1C*	-1.01	2.95E-03
RP11-115L11.1	1.27	1.28E-04	*TRPT1*	-1.27	3.11E-07	*DSCR8*	-1.27	2.99E-03

HHIP-AS1	1.53	1.65E-04	***RP4-647C14.2***	-1.92	3.11E-07	***CTD-2521M24.6***	-1.07	3.08E-03
ARHGAP29	1.07	1.94E-04	***PRDM6***	-1.62	4.13E-07	***RP11-839G9.1***	-1.22	3.12E-03
AC055723.1	1.30	2.39E-04	***PLXDC2***	-1.41	4.13E-07	***FBXL8***	-1.07	3.40E-03
RP11-152K4.2	1.52	2.69E-04	***MAP3K4***	-1.35	6.16E-07	***ACSS1***	-1.28	3.52E-03
RP11-180C1.1	1.47	3.34E-04	***CFI***	-1.55	6.16E-07	***NDUFB9***	-1.10	3.80E-03
RNU6-951P	1.37	3.93E-04	***RP11-146G7.2***	-1.35	6.39E-07	***MIR378H***	-1.16	4.05E-03
THBS1	1.12	4.15E-04	***RP11-778H2.1***	-1.88	6.48E-07	***FRAS1***	-1.26	4.26E-03
DOK1	1.02	4.97E-04	***RP11-248C1.3***	-1.57	1.04E-06	***AC007182.6***	-1.08	4.40E-03
GPR39	1.46	5.12E-04	***TMEM200B***	-1.08	1.08E-06	***RP11-332K15.1***	-1.24	4.43E-03
MORF4L1P7	1.32	6.05E-04	***TBPL1***	-1.58	1.23E-06	***MLPH***	-1.10	4.53E-03
ELTD1	1.41	6.76E-04	***RP3-428L16.2***	-1.03	1.51E-06	***MCF2L***	-1.04	4.98E-03
CTD-2033D15.1	1.19	7.42E-04	***RP11-862L9.3***	-1.64	1.71E-06	***LINC00896***	-1.05	5.32E-03
RP11-25K19.1	1.19	8.29E-04	***RP11-470B22.1***	-1.88	1.74E-06	***FAM216B***	-1.18	5.40E-03
HAS2	1.11	9.49E-04	***RP11-703I16.1***	-1.03	1.86E-06	***RP11-344F13.1***	-1.24	5.53E-03
RP11-71H9.2	1.39	1.17E-03	***CGNL1***	-1.72	1.91E-06	***ARHGEF3-AS1***	-1.16	5.53E-03
COL8A1	1.04	1.23E-03	***LUM***	-1.29	3.16E-06	***SLC2A5***	-1.15	5.60E-03
GDF5OS	1.06	1.23E-03	***AC011484.1***	-1.32	4.21E-06	***WDR91***	-1.14	5.64E-03
KRT18	1.26	1.28E-03	***RP11-575A19.2***	-1.61	4.37E-06	***CLIC2***	-1.14	5.71E-03
SHANK2-AS1	1.29	1.41E-03	***GPR88***	-1.78	4.68E-06	***MACC1***	-1.19	5.78E-03
RP11-495P10.9	1.31	1.55E-03	***IGSF22***	-1.40	5.54E-06	***RP11-782C8.5***	-1.06	5.79E-03
LINC00565	1.15	1.55E-03	***SMOC2***	-1.59	7.97E-06	***MTUS2-AS1***	-1.22	5.97E-03
DSP	1.07	1.76E-03	***ITGB3***	-1.17	8.20E-06	***CYP11A1***	-1.11	6.66E-03
KCNQ5-AS1	1.32	1.84E-03	***CPB1***	-1.78	8.92E-06	***PIEZO2***	-1.02	7.23E-03
SLC15A1	1.35	1.91E-03	***AC106786.1***	-1.61	9.24E-06	***ABCB5***	-1.19	7.40E-03
KIAA1755	1.33	1.91E-03	***AC092642.1***	-1.76	1.00E-05	***MIR920***	-1.18	7.69E-03
KRT8	1.21	1.97E-03	***AKR1C3***	-1.37	1.81E-05	***RP11-720L2.4***	-1.13	7.69E-03
SLC9C1	1.05	2.00E-03	***CDH23***	-1.26	2.28E-05	***STEAP4***	-1.14	7.74E-03
RP11-495P10.8	1.28	2.22E-03	***HPS3***	-1.54	2.77E-05	***CTD-2062F14.3***	-1.09	7.76E-03
RP11-586K2.1	1.11	2.29E-03	***ZIC1***	-1.42	3.27E-05	***CCDC13-AS1***	-1.03	7.76E-03
RP11-495P10.7	1.30	2.48E-03	***CTD-2206N4.4***	-1.50	3.54E-05	***RP11-253D19.1***	-1.19	7.76E-03
FRMPD4-AS1	1.31	2.48E-03	***CTB-39G8.3***	-1.31	4.15E-05	***HRK***	-1.19	7.86E-03
HAS2-AS1	1.28	2.90E-03	***CTD-2330J20.2***	-1.57	4.84E-05	***RP11-53O19.2***	-1.15	8.13E-03
AC107016.1	1.24	2.94E-03	***MITF***	-1.32	5.05E-05	***VCAM1***	-1.13	8.31E-03
AC011755.1	1.29	2.99E-03	***C7orf49***	-1.25	5.46E-05	***ACYP1***	-1.11	8.32E-03
RP11-62C3.6	1.15	3.07E-03	***RP11-680F20.6***	-1.19	6.07E-05	***RP11-782C8.6***	-1.19	8.39E-03
RP11-567G24.1	1.26	3.09E-03	***CTD-2325A15.5***	-1.33	8.32E-05	***AC009492.1***	-1.18	8.61E-03
METTL15P1	1.04	3.32E-03	***CTA-134P22.2***	-1.62	8.77E-05	***RP11-856M7.1***	-1.15	8.66E-03
MIR137HG	1.28	3.33E-03	***SYTL2***	-1.37	1.01E-04	***RP11-18B3.3***	-1.13	9.01E-03
CT62	1.26	3.52E-03	***RP11-993B23.3***	-1.10	1.06E-04	***CYP27A1***	-1.18	9.18E-03
CDH6	1.15	3.66E-03	***IL12A-AS1***	-1.17	1.09E-04	***HCG4P7***	-1.17	9.40E-03
AC131571.2	1.26	3.66E-03	***ADCY8***	-1.61	1.11E-04	***TPRG1-AS2***	-1.08	9.52E-03
RP11-298D21.3	1.18	3.94E-03	***CCDC152***	-1.31	1.14E-04	***AC079210.1***	-1.09	9.69E-03

ABCA4	1.05	4.41E-03	***RSPO2***	-1.34	1.21E-04	***AC016292.1***	-1.16	1.03E-02
DOCK2	1.10	5.01E-03	***CREB5***	-1.16	1.22E-04	***CCRL2***	-1.08	1.04E-02
SCN3A	1.24	5.26E-03	***NDNF***	-1.58	1.39E-04	***CTD-2270N23.1***	-1.01	1.07E-02
LINC00941	1.16	5.54E-03	***SIX3***	-1.57	1.50E-04	***MED16***	-1.04	1.10E-02
FAM196B	1.09	6.37E-03	***TSTD2***	-1.04	1.53E-04	***RTTN***	-1.15	1.15E-02
AC012668.1	1.21	6.63E-03	***PLK1S1***	-1.17	1.65E-04	***AC074366.3***	-1.05	1.15E-02
SERPINE1	1.04	6.69E-03	***GOLGA7B***	-1.57	1.73E-04	***CFTR***	-1.12	1.21E-02
RP11-1090M7.1	1.17	6.69E-03	***CTSK***	-1.26	1.81E-04	***RP11-856M7.2***	-1.00	1.26E-02
RP11-697E14.2	1.05	6.92E-03	***RSPO3***	-1.17	1.81E-04	***RP11-217B7.2***	-1.06	1.36E-02
IQGAP2	1.19	6.99E-03	***RP11-260E18.1***	-1.23	1.98E-04	***RP11-647P12.1***	-1.02	1.43E-02
BIRC3	1.03	7.13E-03	***ENKD1***	-1.15	2.03E-04	***CTD-2540B15.7***	-1.09	1.70E-02
RP11-543N12.1	1.02	7.75E-03	***RP11-304F15.5***	-1.35	2.03E-04	***RP11-1020M18.10***	-1.10	1.72E-02
AC002480.4	1.19	7.76E-03	***RP11-111E14.1***	-1.11	2.05E-04	***SH2D5***	-1.10	1.74E-02
SLC38A5	1.14	7.84E-03	***RAB27B***	-1.27	2.47E-04	***LZTS1-AS1***	-1.09	1.80E-02
AC013463.2	1.18	8.86E-03	***RP11-486P11.1***	-1.40	2.54E-04	***LL22NC03-32F9.1***	-1.07	1.80E-02
RN7SL471P	1.18	9.01E-03	***MTUS1***	-1.50	2.66E-04	***CLCA2***	-1.09	1.81E-02
EPHA5	1.13	9.16E-03	***RP11-97N19.2***	-1.44	2.66E-04	***GPR157***	-1.09	1.82E-02
ESM1	1.11	1.00E-02	***LIMK2***	-1.06	2.69E-04	***LRP1B***	-1.09	1.83E-02
RP5-1046G13.2	1.08	1.12E-02	***KYNU***	-1.51	2.78E-04	***ALDH1A2***	-1.03	1.97E-02
AC069155.1	1.11	1.14E-02	***AP001422.3***	-1.19	2.81E-04	***RP11-13P5.2***	-1.06	2.04E-02
CTD-2547L24.4	1.02	1.31E-02	***MXRA5***	-1.43	2.83E-04	***AL121895.1***	-1.02	2.17E-02
SLIT2	1.04	1.33E-02	***ANGPTL2***	-1.19	3.21E-04	***POLR2E***	-1.07	2.21E-02
RP11-408P14.1	1.07	1.38E-02	***SYNPO2***	-1.20	3.32E-04	***CTD-2540B15.11***	-1.02	2.21E-02
RP11-77K12.4	1.08	1.44E-02	***ITGB8***	-1.11	3.34E-04	***RNFT2***	-1.06	2.40E-02
MESTP3	1.11	1.64E-02	***PPP5C***	-1.18	3.60E-04	***PLEKHF2***	-1.05	2.43E-02
NDST3	1.10	1.72E-02	***RP5-1027G4.3***	-1.49	3.89E-04	***AC005392.13***	-1.05	2.44E-02
MYH3	1.10	1.75E-02	***GRTP1-AS1***	-1.44	3.89E-04	***KDM6A***	-1.01	2.47E-02
RP11-88H10.2	1.10	1.80E-02	***RP4-777D9.2***	-1.07	3.89E-04	***A2M-AS1***	-1.06	2.53E-02
RP11-357J22.1	1.10	1.83E-02	***DMKN***	-1.17	3.93E-04	***TPRG1***	-1.00	2.58E-02
RP13-631K18.5	1.09	1.87E-02	***ARHGAP20***	-1.25	4.01E-04	***RP11-20G13.3***	-1.04	2.69E-02
KCNQ5	1.09	1.89E-02	***RP11-299G20.2***	-1.48	4.12E-04	***SCG2***	-1.00	2.69E-02
RP11-432B6.3	1.06	1.92E-02	***RP11-532M24.1***	-1.31	4.24E-04	***RP13-228J13.8***	-1.04	2.92E-02
AC006050.2	1.02	2.07E-02	***NIPSNAP3B***	-1.30	4.60E-04	***SMUG1***	-1.03	3.01E-02
LINC00886	1.00	2.09E-02	***RP11-782C8.2***	-1.11	4.70E-04	***GALNT15***	-1.02	3.30E-02
NPY4R	1.07	2.27E-02	***TRNP1***	-1.37	4.74E-04	***RP11-849F2.8***	-1.01	3.40E-02
RP11-257K9.3	1.03	2.31E-02	***SAT1***	-1.23	5.08E-04	***AL390240.1***	-1.01	3.52E-02

Supplementary Table 2. Co-localization of DMERs and RA-associated genetic variations.

	DMER	SNPs	Genes
**DEG**	chr2:111490150-111875799	chr2:111607832	*ACOXL*
**H3K27ac**	chr11:60905454-60911325	chr11:60906450	*VPS37C*
	chr11:61593461-61597732	chr11:61595564	*FADS1, FADS2, FADS3*
	chr2:30447310-30450850	chr2:30449594	*LBH*
	chr4:4990554-4994942	chr4:4992367	*CYTL1*
	chr5:131428722-131434367	chr5:131430118	*CSF2*
	chr6:138195142-138197342	chr6:138196066	*TNFAIP3, OLIG3*
**H3K27me3**	chr11:61591923-61597403	chr11:61595564	*FADS1, FADS2, FADS3*
**H3K4me3**	chr11:61594155-61599740	chr11:61595564	*FADS1, FADS2, FADS3*
	chr3:27762156-27766045	chr3:27764623	*EOMES*
	chr6:138187206-138191700	chr6:138187498	*TNFAIP3*
	chr9:37036302-37038252	chr9:37037976	*PAX5*

Supplementary Table 3. Epigenetic clusters with DMER significant enriched. (See Methods for statistics used to calculate *q*-values.)

Cluster	Total regions	Overlapped DMERs*	Associated DMGs*	Enriched biological pathways	Enrichment *q*-value	Section	DMERs								
							H3K27ac	H3K4me1	H3K4me3	H3K36me3	H3K27me3	H3K9me3	Open Chrom	DEG	DNA Methyl
**8**	3,975	579	744	47	1.63E-40	II	299	35	51	5	2	0	96	56	35
**5**	2,119	322	408	1	3.85E-26	II	145	14	31	17	5	0	44	40	26
**4**	2,424	332	403	9	1.28E-19	II	157	15	2	9	0	0	62	44	43
**39**	1,629	237	293	-	2.62E-17	II	82	19	10	12	6	1	48	40	19
**32**	1,103	157	223	1	3.53E-11	II	53	19	0	11	1	0	44	16	13
**13**	1,242	171	239	2	6.37E-11	II	78	17	1	10	0	0	37	16	12
**7**	4,853	498	691	56	3.46E-07	II	293	31	10	1	2	0	100	34	27
**34**	2,065	232	318	-	1.71E-06	II	91	28	1	11	1	0	59	24	17
**24**	1,657	175	238	-	6.71E-04	II	74	22	0	0	0	0	45	18	16
**1**	3,521	507	655	25	2.11E-34	I	56	8	377	6	0	0	13	19	28
**18**	917	190	210	-	8.88E-32	I	69	5	39	12	4	0	23	34	4
**3**	1,550	240	300	-	7.61E-21	I	63	7	113	7	1	0	17	21	11
**2**	3,217	356	463	1	1.61E-08	I	96	13	169	8	1	0	23	23	23

* Cutoff for DMERs from ChIP-seq mark is *q*-value < 0.01, and number of DMGs in each cluster is > 200.

Supplementary Table 4. Differentially modified biological pathways enriched in eight of the 13 clusters (DMERs from ChIP-seq and ATAC-seq are with cutoff q-value<0.01; B-H p-values are calculated by IPA).

Cluster_8

**Ingenuity Canonical Pathways**	**-log(B-H *p*-value)**	**Ratio**
Axonal Guidance Signaling	3.18	0.09
Amyotrophic Lateral Sclerosis Signaling	3.18	0.15
Thyroid Cancer Signaling	2.85	0.23
Leukocyte Extravasation Signaling	2.55	0.10
Protein Kinase A Signaling	2.55	0.08
RhoA Signaling	2.55	0.13
Hepatic Fibrosis / Hepatic Stellate Cell Activation	2.53	0.11
Dopamine-DARPP32 Feedback in cAMP Signaling	2.33	0.11
Clathrin-mediated Endocytosis Signaling	2.18	0.10
Role of Macrophages, Fibroblasts and Endothelial Cells in Rheumatoid	2.17	0.08
Pregnenolone Biosynthesis	1.90	0.40
Cholecystokinin/Gastrin-mediated Signaling	1.88	0.12
Synaptic Long Term Potentiation	1.87	0.11
Phospholipase C Signaling	1.81	0.09
Regulation of the Epithelial-Mesenchymal Transition Pathway	1.74	0.09
Molecular Mechanisms of Cancer	1.74	0.07
Role of NFAT in Cardiac Hypertrophy	1.74	0.09
Role of Osteoblasts, Osteoclasts and Chondrocytes in Rheumatoid Arthritis	1.71	0.08
Signaling by Rho Family GTPases	1.71	0.08
Basal Cell Carcinoma Signaling	1.71	0.13
Histidine Degradation VI	1.71	0.31
Wnt/Ca+ pathway	1.71	0.14
Chondroitin Sulfate Biosynthesis (Late Stages)	1.64	0.16
Adipogenesis pathway	1.64	0.10
Factors Promoting Cardiogenesis in Vertebrates	1.54	0.11
Human Embryonic Stem Cell Pluripotency	1.45	0.09
Nitric Oxide Signaling in the Cardiovascular System	1.45	0.10
Glioblastoma Multiforme Signaling	1.44	0.09
mTOR Signaling	1.42	0.08
Xenobiotic Metabolism Signaling	1.42	0.07
IL-8 Signaling	1.42	0.08
Ubiquinol-10 Biosynthesis (Eukaryotic)	1.40	0.24
Neuropathic Pain Signaling In Dorsal Horn Neurons	1.39	0.10
Chondroitin Sulfate Biosynthesis	1.39	0.13
CREB Signaling in Neurons	1.39	0.08
Huntington's Disease Signaling	1.38	0.08
Phagosome Formation	1.38	0.10
Gap Junction Signaling	1.38	0.08
Thyroid Hormone Biosynthesis	1.38	0.67
Melatonin Signaling	1.38	0.12
Dermatan Sulfate Biosynthesis	1.38	0.13
Dermatan Sulfate Biosynthesis (Late Stages)	1.37	0.14
Sperm Motility	1.35	0.09
Calcium Signaling	1.35	0.08
fMLP Signaling in Neutrophils	1.32	0.09
Production of Nitric Oxide and Reactive Oxygen Species in Macrophages	1.30	0.08
ILK Signaling	1.30	0.08

Cluster_1

**Ingenuity Canonical Pathways**	**-log(B-H *p*-value)**	**Ratio**

Protein Kinase A Signaling	2.66	0.08
Phospholipase C Signaling	2.66	0.09
p53 Signaling	2.62	0.13
Retinoic acid Mediated Apoptosis Signaling	1.96	0.16
Apoptosis Signaling	1.96	0.13
RhoA Signaling	1.96	0.11
RhoGDI Signaling	1.96	0.09
Hypoxia Signaling in the Cardiovascular System	1.96	0.14
CCR3 Signaling in Eosinophils	1.95	0.10
Androgen Signaling	1.95	0.11
Sumoylation Pathway	1.95	0.12
Unfolded protein response	1.95	0.15
fMLP Signaling in Neutrophils	1.71	0.10
nNOS Signaling in Neurons	1.67	0.15
Calcium-induced T Lymphocyte Apoptosis	1.67	0.13
Death Receptor Signaling	1.67	0.11
Nitric Oxide Signaling in the Cardiovascular System	1.67	0.10
Adipogenesis pathway	1.48	0.09
Nur77 Signaling in T Lymphocytes	1.46	0.13
VEGF Signaling	1.46	0.10
Pancreatic Adenocarcinoma Signaling	1.46	0.09
UVA-Induced MAPK Signaling	1.46	0.10
Regulation of Cellular Mechanics by Calpain Protease	1.41	0.13
Huntington's Disease Signaling	1.38	0.07
Breast Cancer Regulation by Stathmin1	1.31	0.07

Cluster_5

Ingenuity Canonical Pathways	-log(B-H *p*-value)	Ratio
Dendritic Cell Maturation	1.41	0.07

Cluster_4

Ingenuity Canonical Pathways	-log(B-H *p*-value)	Ratio
Integrin Signaling	2.58	0.08
Bladder Cancer Signaling	1.95	0.11
Clathrin-mediated Endocytosis Signaling	1.64	0.07
Paxillin Signaling	1.57	0.08
Agrin Interactions at Neuromuscular Junction	1.57	0.10
Regulation of the Epithelial-Mesenchymal Transition Pathway	1.57	0.06
HGF Signaling	1.57	0.08
Caveolar-mediated Endocytosis Signaling	1.57	0.10
Ephrin Receptor Signaling	1.49	0.06

Cluster_32

Ingenuity Canonical Pathways	-log(B-H *p*-value)	Ratio
Huntington's Disease Signaling	2.28	0.05

Cluster_13

Ingenuity Canonical Pathways	-log(B-H *p*-value)	Ratio
GNRH Signaling	1.60	0.06
Huntington's Disease Signaling	1.60	0.05

Cluster_2

Ingenuity Canonical Pathways	-log(B-H *p*-value)	Ratio
Dopamine-DARPP32 Feedback in cAMP Signaling	1.48	0.08

Cluster_7

Ingenuity Canonical Pathways	-log(B-H *p*-value)	Ratio

Human Embryonic Stem Cell Pluripotency	5.22	0.15
Axonal Guidance Signaling	3.84	0.08
Role of Osteoblasts, Osteoclasts and Chondrocytes in Rheumatoid Arthritis	3.24	0.10
Ovarian Cancer Signaling	3.03	0.12
Protein Kinase A Signaling	2.42	0.08
Wnt/β-catenin Signaling	2.42	0.10
G-Protein Coupled Receptor Signaling	2.42	0.09
Urate Biosynthesis/Inosine 5'-phosphate Degradation	2.42	0.36
CDK5 Signaling	2.19	0.12
Gap Junction Signaling	2.06	0.10
Regulation of the Epithelial-Mesenchymal Transition Pathway	1.99	0.09
Ephrin Receptor Signaling	1.97	0.09
Purine Nucleotides Degradation II (Aerobic)	1.97	0.26
Glioblastoma Multiforme Signaling	1.97	0.09
Epithelial Adherens Junction Signaling	1.97	0.10
p53 Signaling	1.97	0.11
Macropinocytosis Signaling	1.97	0.12
Clathrin-mediated Endocytosis Signaling	1.97	0.09
FAK Signaling	1.92	0.11
Basal Cell Carcinoma Signaling	1.92	0.13
Myc Mediated Apoptosis Signaling	1.89	0.13
Role of Macrophages, Fibroblasts and Endothelial Cells in Rheumatoid	1.89	0.07
Guanosine Nucleotides Degradation III	1.89	0.31
Leukocyte Extravasation Signaling	1.89	0.08
Virus Entry via Endocytic Pathways	1.89	0.11
Role of NANOG in Mammalian Embryonic Stem Cell Pluripotency	1.84	0.10
Mouse Embryonic Stem Cell Pluripotency	1.81	0.11
ILK Signaling	1.81	0.08
PCP pathway	1.77	0.13
Nitric Oxide Signaling in the Cardiovascular System	1.77	0.10
Cellular Effects of Sildenafil (Viagra)	1.77	0.10
Estrogen-Dependent Breast Cancer Signaling	1.77	0.12
Amyotrophic Lateral Sclerosis Signaling	1.75	0.10
Paxillin Signaling	1.75	0.10
Hepatic Fibrosis / Hepatic Stellate Cell Activation	1.73	0.08
Adenosine Nucleotides Degradation II	1.68	0.25
eNOS Signaling	1.68	0.09
Colorectal Cancer Metastasis Signaling	1.68	0.07
Thyroid Cancer Signaling	1.67	0.15
Role of Tissue Factor in Cancer	1.56	0.09
PTEN Signaling	1.56	0.09
Integrin Signaling	1.55	0.08
HER-2 Signaling in Breast Cancer	1.50	0.10
NF-κB Signaling	1.50	0.08
Thyroid Hormone Biosynthesis	1.50	0.67
Corticotropin Releasing Hormone Signaling	1.46	0.09
MSP-RON Signaling Pathway	1.45	0.12
Telomerase Signaling	1.45	0.09
Glioma Signaling	1.43	0.09
Reelin Signaling in Neurons	1.43	0.10
Neurotrophin/TRK Signaling	1.43	0.11
Ceramide Signaling	1.42	0.10
NAD Salvage Pathway II	1.39	0.19
Melanocyte Development and Pigmentation Signaling	1.38	0.09
Relaxin Signaling	1.38	0.08
HGF Signaling	1.35	0.09

Supplementary Table 5. Known motifs identified in 13 DMER significantly enriched clusters. (Due to small input size, q-value (calculated by HOMER) < 0.1 was used as the cutoff).

Cluster_8

Motif	Consensus	*q*-value	% of Target	% of Background
Atf3(bZIP)	DATGASTCATHN	0.012	10.39%	5.05%
ZBTB18(Zf)	AACATCTGGA	0.020	9.83%	4.96%
BMYB(HTH)	NHAACBGYYV	0.020	19.10%	12.16%
BATF(bZIP)	DATGASTCAT	0.023	9.55%	4.97%
AP-1(bZIP)	VTGACTCATC	0.023	10.67%	5.88%
MYB(HTH)	GGCVGTTR	0.037	20.79%	14.34%
JunB(bZIP)	RATGASTCAT	0.037	8.15%	4.21%
NF1(CTF)	CYTGGCABNSTGCCAR	0.039	7.87%	4.09%
HIF-1b(HLH)	RTACGTGC	0.079	14.33%	9.45%
AMYB(HTH)	TGGCAGTTGG	0.079	17.98%	12.59%
Bach2(bZIP)	TGCTGAGTCA	0.079	3.65%	1.43%

Cluster_5

Motif	Consensus	*q*-value	% of Target	% of Background
Atf3(bZIP)	DATGASTCATHN	0.065	12.38%	5.59%
AP-1(bZIP)	VTGACTCATC	0.065	13.37%	6.53%
Pitx1:Ebox(Home	YTAATTRAWWCCAGATGT	0.065	5.45%	1.61%
Fra2(bZIP)	GGATGACTCATC	0.084	8.91%	3.86%

Cluster_4

Motif	Consensus	*q*-value	% of Target	% of Background
Fra2(bZIP)	GGATGACTCATC	0.001	11.86%	3.91%
Atf3(bZIP)	DATGASTCATHN	0.001	14.95%	5.79%
AP-1(bZIP)	VTGACTCATC	0.001	15.46%	6.50%
Fra1(bZIP)	NNATGASTCATH	0.002	12.37%	4.75%
JunB(bZIP)	RATGASTCAT	0.003	11.86%	4.63%
SPDEF(ETS)	ASWTCCTGBT	0.010	16.49%	8.36%
BATF(bZIP)	DATGASTCAT	0.017	12.37%	5.71%
ETS:RUNX(ETS,	RCAGGATGTGGT	0.023	3.61%	0.71%

Cluster_39

Motif	Consensus	*q*-value	% of Target	% of Background
BATF(bZIP)	DATGASTCAT	0.008	14.48%	5.19%
Fra1(bZIP)	NNATGASTCATH	0.009	12.41%	4.26%
Atf3(bZIP)	DATGASTCATHN	0.013	13.79%	5.37%
AP-1(bZIP)	VTGACTCATC	0.020	14.48%	6.12%
Fra2(bZIP)	GGATGACTCATC	0.020	10.34%	3.62%
JunB(bZIP)	RATGASTCAT	0.034	11.03%	4.31%

Cluster_32

Motif	Consensus	*q*-value	% of Target	% of Background
AP-1(bZIP)	VTGACTCATC	0.000	23.96%	6.69%
Jun-AP1(bZIP)	GATGASTCATCN	0.003	10.42%	1.88%
JunB(bZIP)	RATGASTCAT	0.003	16.67%	4.85%
BATF(bZIP)	DATGASTCAT	0.003	17.71%	5.80%
Fosl2(bZIP)	NATGASTCABNN	0.003	11.46%	2.61%
Atf3(bZIP)	DATGASTCATHN	0.004	17.71%	6.04%
Fra1(bZIP)	NNATGASTCATH	0.004	15.62%	4.91%
Bach2(bZIP)	TGCTGAGTCA	0.005	8.33%	1.52%
Fra2(bZIP)	GGATGACTCATC	0.006	13.54%	4.10%

JunD(bZIP)	ATGACGTCATCN	0.010	5.21%	0.59%
NF1-Nanog(Homeobox	YTGCCAAG	0.023	30.21%	16.60%
	RGCCATTAAC	0.044	59.38%	43.72%
ZNF136(Zf)	YTKGATAHAGTATTCTWG	0.097	6.25%	1.51%
NPAS(bHLH)	NVCACGTG	0.099	27.08%	15.95%

Cluster_7

Motif	Consensus	*q*-value	% of Target	% of Background
TEAD(TEA)	YCWGGAATGY	0.004	14.67%	7.69%
Fra2(bZIP)	GGATGACTCATC	0.008	8.98%	4.02%
BATF(bZIP)	DATGASTCAT	0.008	11.38%	5.71%
Jun-AP1(bZIP)	GATGASTCATCN	0.008	5.39%	1.86%
Fosl2(bZIP)	NATGASTCABNN	0.008	6.59%	2.58%
AP-1(bZIP)	VTGACTCATC	0.008	12.57%	6.80%
TEAD4(TEA)	CCWGGAATGY	0.013	14.67%	8.72%
JunB(bZIP)	RATGASTCAT	0.015	9.28%	4.74%
Bach2(bZIP)	TGCTGAGTCA	0.015	4.49%	1.59%
Atf3(bZIP)	DATGASTCATHN	0.015	10.78%	5.89%
TEAD2(TEA)	CCWGGAATGY	0.015	10.48%	5.68%
JunD(bZIP)	ATGACGTCATCN	0.015	2.40%	0.53%
Atf4(bZIP)	MTGATGCAAT	0.015	6.59%	2.99%
Stat3+il21(Stat)	SVYTTCCNGGAARB	0.022	10.78%	6.15%
RUNX-	GCTGTGGTTW	0.022	11.08%	6.39%
STAT4(Stat)	NYTTCCWGGAAR	0.030	14.37%	9.17%
Stat3(Stat)	CTTCCGGGAA	0.030	8.08%	4.29%
Fra1(bZIP)	NNATGASTCATH	0.030	8.68%	4.74%
Chop(bZIP)	ATTGCATCAT	0.030	5.39%	2.43%
STAT5(Stat)	RTTTCTNAGAAA	0.051	6.29%	3.19%
NFAT:AP1(RHD,	SARTGGAAAAWRTGAGTC	0.051	4.19%	1.76%
RUNX(Runt)	SAAACCACAG	0.062	9.88%	6.00%
MITF(bHLH)	RTCATGTGAC	0.063	12.57%	8.21%
Atf2(bZIP)	NRRTGACGTCAT	0.063	5.99%	3.08%
Lhx3(Homeobox)	ADBTAATTAR	0.063	23.95%	18.08%
IRF2(IRF)	GAAASYGAAASY	0.066	2.69%	0.94%
CEBP:AP1(bZIP)	DRTGTTGCAA	0.068	11.68%	7.58%

Cluster_24

Motif	Consensus	*q*-value	% of Target	% of Background
JunB(bZIP)	RATGASTCAT	0.002	16.51%	4.92%
Fra2(bZIP)	GGATGACTCATC	0.005	13.76%	3.94%
BATF(bZIP)	DATGASTCAT	0.010	16.51%	6.00%
Atf3(bZIP)	DATGASTCATHN	0.010	16.51%	6.07%
Fra1(bZIP)	NNATGASTCATH	0.010	14.68%	4.97%
Fosl2(bZIP)	NATGASTCABNN	0.010	10.09%	2.51%
AP-1(bZIP)	VTGACTCATC	0.010	17.43%	6.89%
Bach2(bZIP)	TGCTGAGTCA	0.040	6.42%	1.39%

Cluster_18

Motif	Consensus	*q*-value	% of Target	% of Background
RUNX(Runt)	SAAACCACAG	0.000	18.02%	5.09%
RUNX-	GCTGTGGTTW	0.034	14.41%	5.15%
RUNX2(Runt)	NWAACCACADNN	0.034	15.32%	5.73%
RUNX1(Runt)	AAACCACARM	0.046	17.12%	7.40%
