# Supplementary material for: Comprehensive epigenetic landscape of rheumatoid arthritis fibroblast-like synoviocytes
Source: Nat Commun. 2018 May 15;9:1921. doi: 10.1038/s41467-018-04310-9 (PMC5953939; doi:10.1038/s41467-018-04310-9)
Supplement: Supplementary file 2 — Description of Additional Supplementary Files [file 41467_2018_4310_MOESM2_ESM.pdf]

Description of Additional Supplementary Files

File Name: Supplementary Data 1
Description: DMERs with fold changes and q values. (See Methods for statistics used to calculate q values.)
